# Recent Advances in Deep Learning-Based Source Camera Identification and Device Linking

**DOI:** 10.3390/s25247432

**Published:** 2025-12-06

**Authors:** Zimeng Li, Ngai-Fong Law

**Affiliations:** Department of Electrical and Electronic Engineering, The Hong Kong Polytechnic University, Hong Kong, China; li-zimeng.li@connect.polyu.hk

**Keywords:** sensor artefacts, photo-response non-uniformity, camera identification, device linking

## Abstract

Photo-response non-uniformity (PRNU) has long been regarded as a reliable method for source camera identification and device linking in forensic applications. Recent advances in deep learning (DL) have introduced diverse architectures, including convolutional neural networks, residual learning, encoder–decoder representations, dual-branch structures, and contrastive learning, to capture specific sensor artifacts. This review summarizes the performance of these DL techniques across both tasks and compares their effectiveness at the model and device levels over time. While DL approaches achieve strong model-level accuracy, robust device-level identification remains challenging, particularly in modern imaging pipelines that involve camera-integrated or AI-driven enhancements during capture. These findings underscore the need for improved techniques and updated datasets to address evolving photograph capture practices.

## 1. Introduction

The widespread use of digital images in entertainment, social networking, and legal contexts has highlighted the importance of accurately identifying the source camera that captured them. This issue typically arises in two distinct scenarios: (1) verifying the source of an image among several potential cameras and (2) determining whether the same camera took two images. The former is known as source camera identification [1], while the latter is referred to as the device linking problem [2]. Source camera identification is crucial when the set of potential source cameras is known, framing the task as a multi-class classification problem. Conversely, device linking is particularly relevant for verifying associations between online accounts by determining whether their profile images originate from the same camera.

Both source camera identification and device linking usually rely on extracting a unique sensor noise pattern, known as photo-response non-uniformity (PRNU). This statistical pattern is inherent to each camera sensor and is present in every image produced by that sensor [1]. By analyzing PRNU, it becomes possible to determine whether a photograph originated from a specific camera or whether the same device captured the two images.

Traditionally, signal processing-based filtering methods have been used to extract sensor noise from photographs. Although introduced in 2006 [1], the sensor noise-based approach remains widely accepted in forensic and legal contexts for verifying the authenticity of image sources. The emergence of deep learning (DL) techniques has introduced new possibilities for camera identification. Researchers have explored popular architectures such as residual networks, U-Net, and ConvNet for source camera identification. In this context, the task is formulated as a classification problem, where deep learning models are trained to learn forensic features directly from image data, thereby differentiating images from a predefined set of cameras. These methods often address identification at three levels:

- Camera brand identification: identifying the brand of the camera that captured the photograph.
- Camera model identification: identifying the camera model that captured the photograph.
- Camera device identification: identifying the exact device that took the photograph.

Since the first deep learning-based study in 2016 [3], research has consistently shown that while deep learning methods excel at distinguishing camera brands and models, device-level identification remains challenging and requires further investigation. These methods also struggle when encountering previously unseen cameras not included in the training data. Moreover, deep learning approaches typically require a large number of images for training, which may not be feasible in forensic scenarios where only a limited number of images are available.

This constraint becomes particularly critical in device linking tasks, where the goal is to determine whether two images—often the only ones available—were captured by the same camera. In such cases, deep learning methods also attempt to extract camera-specific information from images. Two main approaches have been explored in the literature: one formulates the problem as a few-shot learning task, while the other employs Siamese networks and contrastive learning frameworks to measure similarity between camera-specific artefacts in image pairs. The weak nature of these artefacts makes the linking process particularly challenging.

The primary purpose of this review is to examine state-of-the-art deep learning-based methods for source camera identification and device linking, with a clear distinction between model-level and device-level performance. Recent reviews have primarily focused on source camera identification, leaving device linking comparatively underexplored despite its relevance in forensic and social media contexts. Specifically, prior reviews can be summarized as follows:

- Ref. [4] surveyed works published before 2021 on source identification using noise patterns in machine learning-based systems.
- Ref. [5] focused on PRNU and related techniques such as lens radial distortion, color filter array interpolation, and auto-white balance approximation.
- Ref. [6] reviewed PRNU, statistical methods, and deep learning methods in classification settings. While it mentions model and device-level identification, it lacks performance and comparative analysis.
- Ref. [7] explored PRNU, CNN-based, feature-based, and metadata-based methods, questioning whether PRNU remains the gold standard in modern imaging pipelines.
- Ref. [8] provided an overview of camera noise types and neural network-based noise estimation techniques. However, it lacks performance analysis of identification.

**Figure 1:**
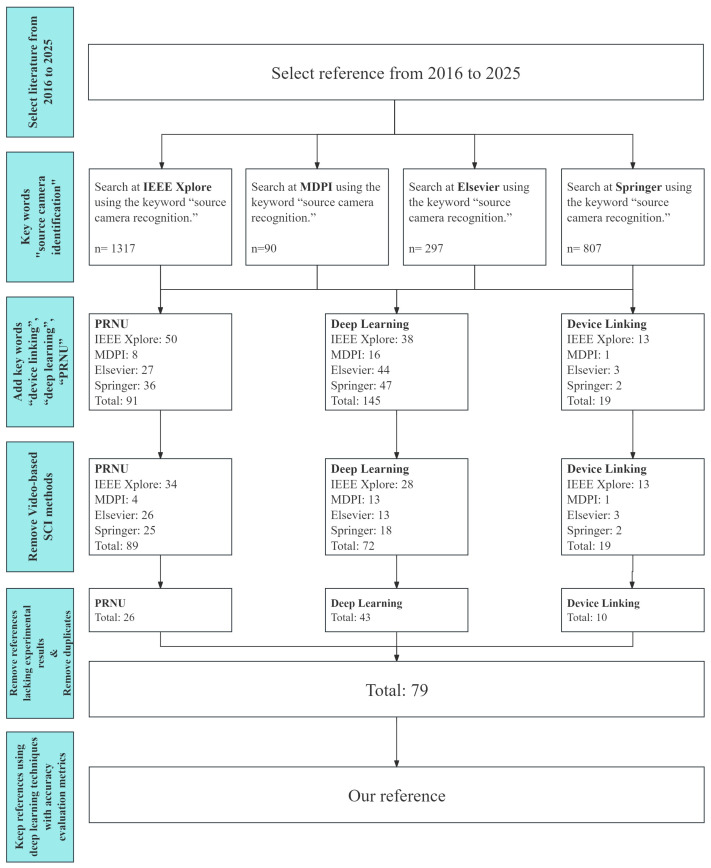
Search strategy for selecting studies on DL-based source camera identification and device linking.

This review aims to evaluate the performance of state-of-the-art DL-based methods and compare them with the PRNU-based methods. We begin by reviewing traditional PRNU-based approaches, given their established role and legal relevance in forensic contexts. Then, we examine deep learning methods for both source camera identification and device linking developed between 2016 and 2025. As shown in Figure 1, a search was conducted across major journals, conference proceedings, and digital libraries (IEEE Xplore, Springer, Elsevier, MDPI) using keywords such as “source camera identification”, “device linking”, “deep learning”, “PRNU”, and “camera attribution”. Studies were included if they proposed DL-based methods for source camera identification or device linking, provided comprehensive experimental validation, and reported performance metrics at either the model or device levels. Works lacking experimental results or focusing on video-based identification were excluded. From approximately 180 papers initially identified, 30 met all criteria, with 16 published on or after 2022. Their performance was analyzed at both the model and device levels. Through this, we aim to assess the achievements of deep learning methods in comparison to traditional PRNU-based approaches, highlighting their strengths, limitations, and practical implications.

Modern cameras are equipped with automatic settings that enhance photographs in real-time during image capture, often complemented by AI-driven photo enhancement features. These developments may modify the extracted sensor noise and camera-specific artefacts, potentially affecting identification accuracy. However, most available datasets do not account for these effects, making it difficult to systematically assess their impact on source camera identification and device linking. As discussed in [7], the modern imaging pipeline introduces unique challenges for PRNU-based methods. Currently, no study has examined whether DL-based methods suffer from similar issues. This review addresses this critical issue by evaluating the impact of the modern imaging pipelines on DL-based methods. Given the increasing use of AI-enhanced photography, this review provides timely insights for forensic analysts and researchers.

The paper is structured as follows: Section 2 examines sensor noise-based methods and their performance on both source camera identification and device linking tasks. Subsequently, Section 3 and Section 4 explore deep learning methods for source camera identification and device linking, respectively. Section 5 compares the performance of these methods, while Section 6 discusses the challenges associated with them. Finally, Section 7 concludes the paper.

## 2. Sensor Noise-Based Methods for Source Camera Identification and Device Linking

Source camera identification typically involves two steps: (1) extracting camera-specific features and (2) verifying their presence in a test image. One of the most widely used features in source camera identification is PRNU (photo-response non-uniformity) noise, which arises from manufacturing imperfections in the camera sensor. Since this noise pattern originates from hardware defects, it is unique to each camera device [1,9]. Mathematically, PRNU can be extracted using denoising techniques, as shown below:

(1) $$w_{i}=I_{i}-D\left(I_{i}\right)=I_{i}K+\varepsilon$$

where $I_{i}$ represents the i-th image from a specific camera, D(·) denotes the denoising operator, $w_{i}$ is the noise residue of the i-th image, *K* is the camera PRNU, and *ε* is the additive noise component. Denoising can be achieved through wavelet-based denoising, BM3D [10], or the recently proposed optimal filters [11]. Due to additive noise and the inherently weak nature of the PRNU, it is more reliable to estimate the camera’s PRNU using multiple smooth content images captured by the same device, applying either simple averaging [1] or maximum likelihood estimation [9]. This camera’s PRNU can then be considered as its camera signature.

To determine whether a specific camera took an image, its noise residue is extracted using (1) and then compared with the camera PRNU. Metrics such as normalized cross correlation and peak correlation energy (*PCE*) [2,12,13] can be used to assess the similarity between the noise residue and the PRNU. A threshold value is required to act as a decision rule in digital camera identification [14]. Most research uses *PCE* because it is independent of image size. The *PCE* between x and y is defined as

(2) $$PCE(x,y)=\frac{\rho {\left(s_{peak}=0,x,y\right)}^{2}}{\frac{1}{MN-\left|A\right|}\sum_{s\notin A}\rho {\left(s,x,y\right)}^{2}}$$

where ρ(s,x,y) is the dot product between $x-\bar{x}$ and $y(s)-\bar{y}$, y(s) is obtained by circularly shifting y by a two-dimensional vector s. The bar over the symbol represents its mean value, *A* is a small neighborhood around the peak, and *M* and *N* are the width and height of the image, respectively.

In practice, test images may contain complex structures that introduce scene content artefacts into the noise residue, thereby degrading the performance of source camera identification. Various approaches have been proposed to address issues with scene content artefacts. For example, a strong component in the noise residue indicates that it is less trustworthy and should be attenuated [15]. In [16], both the camera PRNU and the noise residue were obtained by removing the magnitude of the noise residue in the frequency domain and averaging the phase component only. To further improve accuracy and compensate for scene content issues, region reliability is estimated to produce a weighted correlation value [17,18]. A guided filtering approach is proposed in [19] to enhance the PRNU by removing interference from low-frequency components. Table 1 shows the performance of the PRNU-based source device identification. With the use of fifty smooth images to construct the camera PRNU, all these methods can achieve high identification accuracy. The true-positive rates remain satisfactory even at very low false-positive rates. Due to its high accuracy and low false-positive rate, the PRNU-based method remains widely accepted by courts for verifying image sources [19].

The PRNU-based method is effective when there are enough smooth images to construct the camera PRNU. In device linking, the camera PRNU cannot be estimated because only two images are available for estimation. Direct comparison between the two noise residues is required to perform device linking. It is essential to select plain and bright image regions for comparing the similarity between the two images [20]. Table 2 shows the performance of the PRNU-based method under this situation. Despite achieving high accuracy and a low false-positive rate, the true-positive rate is notably low. This means that most image pairs are considered not to have originated from the same camera device. Results in Table 2 indicate the difficulty of reliably relating the source of two images based on sensor noise.

Since its introduction in 2006 [1], the PRNU-based method has served as a benchmark for forensic comparisons. It is a court-accepted method with documented error rates: a false-negative rate of 2.38% and a false-positive rate of 0.0024% [21]. Recent studies [7,21] have focused on evaluating the reliability of PRNU-based methods in forensic image analysis, particularly under varying image conditions. For example, reference [21] shows that image brightness could affect error rates. Overexposed and underexposed images tend to have lower true-positive rates than bright images. This means that the PRNU-based methods may miss more images from the suspect camera.

Photo-response non-uniformity (PRNU) is highly sensitive to geometric transformations such as rotation and scaling [22,23,24]. These operations alter the spatial alignment of sensor noise patterns, which PRNU-based algorithms rely on for camera identification [25,26,27]. Even minor geometric changes can significantly degrade correlation scores, leading to false negatives in source camera identification [25]. This vulnerability poses a significant challenge in real-world forensic scenarios. To mitigate this, techniques such as robust feature extraction and watermarking have been considered to preserve device-level forensic traces in the presence of geometric attacks [26,27,28]. However, achieving robustness remains an open research problem.

## 3. Deep Learning Approaches for Source Camera Identification

**Figure 2:**
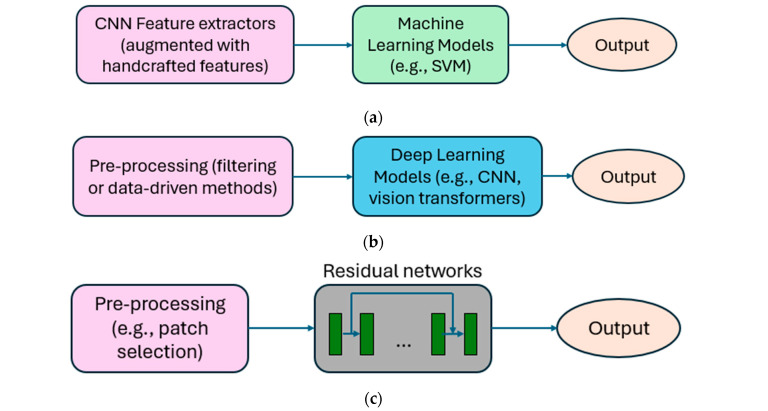

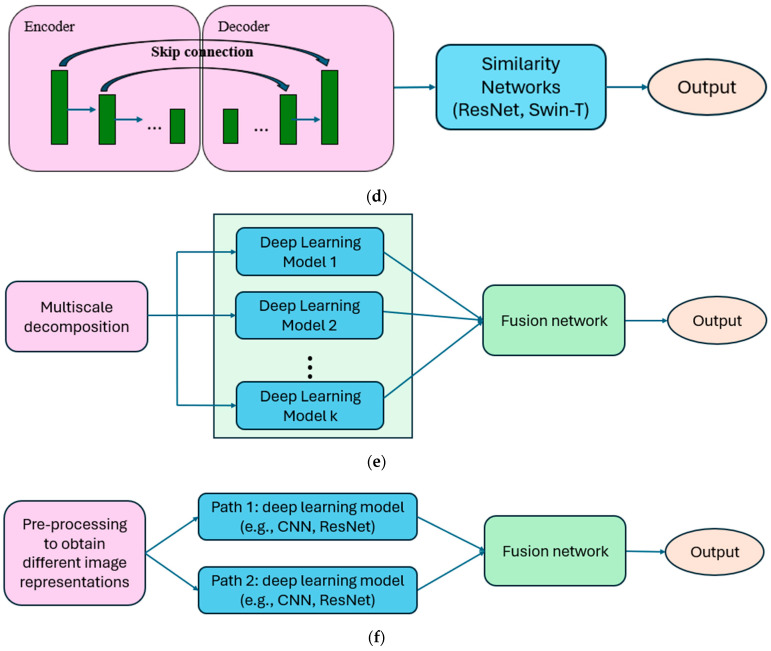
Schematic diagrams of (**a**) CNN feature extraction-based methods, (**b**) pre-processing-based methods, (**c**) residual-based methods, (**d**) encoder–decoder-based methods, (**e**) multiscale-based methods, and (**f**) dual-path-based methods.

While PRNU-based methods have demonstrated high accuracy and legal credibility, their performance can degrade under challenging conditions such as overexposure, underexposure, and complex scenes [15,16,17,18,19]. These limitations have motivated researchers to explore deep learning techniques, which aim to extract forensic features directly from images in a data-driven manner. This section reviews the evolution of deep learning techniques in this domain, with a focus on architectural developments in source camera identification. Table 3 presents a chronological overview of deep learning techniques proposed for source camera identification from 2016 to 2025, while Figure 2 summarizes their key design components.

Early studies employed convolutional neural network (CNN) architectures to extract forensic features from images. As illustrated in Figure 2a, these features were fed into a classical machine learning model for classification. For example, in [3], a CNN was used to extract camera features, which were subsequently classified using a support vector machine (SVM). This work demonstrated the effectiveness of CNN models on camera model identification for the first time. More recent developments combined spectral features from wavelet transforms with spatial features from local binary patterns, using multiclass models such as SVM, LDA, and k-NN for classification [29].

Since image scene information is generally irrelevant to camera identification, various pre-processing techniques have been developed to suppress scene content and enhance the extraction of relevant camera features, as illustrated in Figure 2b. For example, researchers have considered fixed high-pass filters, wavelet-based denoising filters [30], and median filters [31]. An adaptive constraint convolution layer was introduced in [32] to suppress image content. The pre-processing module in [33] consisted of an edge map extraction followed by low-pass filtering. In contrast to these fixed filters, reference [34] employed a data-driven pre-processing block to remove irrelevant content from input images dynamically. Recent advancements involve extracting angular and radial image features to capture pixel variations and relationships within neighborhoods [35]. These features are then integrated into vision transformers for classification. All these pre-processing techniques have consistently demonstrated superior performance in camera model identification tasks.

Residual networks have gained attention in this domain. Their core principle is to learn residual mappings—the difference between the input and the output of a set of layers, as illustrated in Figure 2c. This approach helps preserve subtle forensic traces in photos and mitigates issues such as vanishing gradients. Content-adaptive fusion residual networks were explicitly designed for small-sized images [36]. The input images were categorized into three subsets based on their image content characteristics: saturation, smoothness, and others. Subsequently, each subset was individually trained on a residual network model. Later, the residual network was enhanced with a domain knowledge-driven pre-processing module [37]. A hierarchical multi-task learning mechanism was adopted for three-level identification: brand-level, model-level, and device-level. This involved introducing a domain knowledge-driven pre-processing module that incorporated multiscale high-pass filters, followed by convolutional layers and ResNet. Additionally, Ref. [38] explored a combination of convolutional layers and residual blocks. They also introduced a technique to identify images from unknown camera models by setting a threshold for output prediction scores, enhancing the network’s capability to handle previously unseen camera sources.

On another front, the U-Net model, known for its encoder–decoder structure, has gained popularity. As shown in Figure 2d, the encoder progressively reduces the spatial dimensions of the input image while capturing camera features, and the decoder reconstructs the image representation by upsampling, restoring spatial resolution, and combining it with the encoder output through skip connections. This design enables the network to retain camera characteristics while learning contextual information. A hierarchical architecture of U-Net with dense connectivity and residual learning was considered for PRNU extraction [39]. A U-Net encoder–decoder unit was designed as a residual noise feature extractor, in which the embedding from the residual noise map was subsequently used for classification [40]. Besides, the camera fingerprint was extracted from a U-Net [41]. Features from different scales of feature maps of the Transformer block were fused using the graph convolutional network.

Multiscale analysis is always crucial in feature extraction across various domains. As illustrated in Figure 2e, an image can first be decomposed into multiple scales to capture global and local information. Building on this concept, a fine-grained, multiscale residual prediction strategy was employed in [42] to mitigate the impact of scene content on source identification. In [43], multiscale filters were used to suppress various scene content. Additionally, in [44], a multiscale encoder–decoder structure was considered, involving the selection of image patches at different scales to construct the camera fingerprint.

All previous works were based on a single-path architecture. The adoption of dual-path networks, as illustrated in Figure 2f, has emerged as a promising strategy to integrate complementary information collected from two distinct branches. For example, a compact dual-path attention-enhanced ConvNeXt network was developed in [45]. A channel attention mechanism was employed to preserve high-frequency residual information, which is crucial for camera fingerprint construction, while minimizing interference from scene content. An adaptive dual-branch fusion network was introduced to extract multiscale features, and a bottleneck residual module was proposed to facilitate the transfer of shallow features for capturing weak camera features [46]. Additionally, a dual-branch CNN-based framework that fused low-level features from color images and high-pass filtered images was introduced to provide complementary features for the identification task [47]. Building on this trend, Ref. [48] introduced a contrastive learning strategy using a heterogeneous dual-branch network to refine the learning of camera fingerprints. Two approaches to extract camera information were employed within the dual-branch framework. Through contrastive learning, the shared forensic features related to the camera model between the two branches are enhanced, effectively filtering out irrelevant scene content information. The camera fingerprint information extracted from both branches is then integrated into a classification module to perform the camera model identification task. The concept of dual-branch architecture is further enhanced in [49] through the integration of multiscale decomposition and wavelet-based feature refinement. By employing multi-level decomposition and fusing features across scales, the method effectively amplifies subtle noise patterns, thereby improving the extraction of forensic traces for source camera identification.

**Table 3:** Chronological overview of deep learning techniques for source camera identification.

Year	Techniques	References
2016	Pre-processing	Highpass [30]
2017	Pre-processing	Median filter [31]
	Pre-processing	Adaptive conv layer [32]
	CNN feature extraction	with SVM [3]
	Residual network	Content-adaptive fusion [36]
2019	Residual network	Domain knowledge [37]
2020	Pre-processing	Edge map [33]
2021	Pre-processing	Data-driven [34]
	Multi-scale	Residual prediction [42]
	Multi-scale	Multiple-scale filters [43]
2022	U-Net	Hierarchical [39]
	Multiscale	Multiscale encoder-decoder [44]
2023	CNN feature extraction	Wavelet with LBP [29]
	U-Net	Residual-noise extraction [40]
2024	Residual network	Conv with residual [38]
	U-Net	Multi-scale with transformer [41]
	Dual-path	ConvNeXt [45]
	Dual-path	Multiscale feature fusion [46]
	Dual-path	High and low pass fusion [47]
2025	Pre-processing	Angular and radial feature extraction [35]
	Dual-path	Contrastive learning [48]
	Dual-path	Multiscale with wavelet [49]

## 4. Deep Learning Approaches for Device Linking

In device linking, the goal is to determine whether two images originate from the same camera. Although the deep learning approaches discussed in Section 3 can be applied to compare noise residues between two images, their performance is limited by the quality of these residues and the presence of other noise components, as illustrated in Equation (1). Consequently, their effectiveness is significantly hindered. Table 4 provides a chronological overview of the deep learning techniques proposed for device linking from 2019 to 2025. Two main strategies have been developed to address the device linking problem:

- Few-shot learning, particularly one-shot learning.
- Contrastive learning, which compares image pairs.

**Figure 3:**
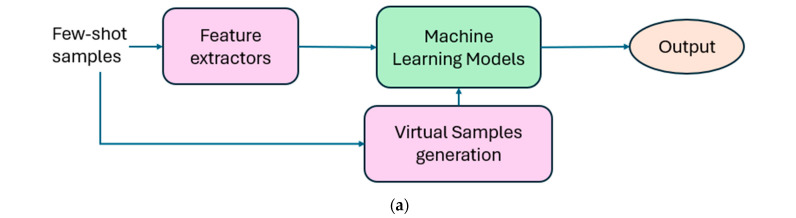

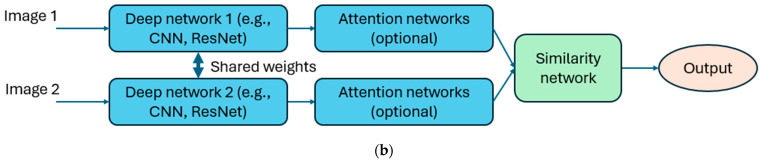
Schematic diagrams of (**a**) few-shot learning-based and (**b**) contrastive learning-based methods.

Their key design strategies are given in Figure 3. In few-shot learning, virtual samples are generated to augment limited training data, and semi-supervised learning strategies are generally employed to improve performance under data-scarce conditions. For example, Ref. [50] proposed generating virtual samples using a global fuzzification and information diffusion strategy. In [51], a distance-based ensemble strategy was combined with a self-correction mechanism for semi-supervised learning. Similarly, Ref. [52] utilized multiple distance measures and coordinate pseudo-label selection in its semi-supervised learning framework. Despite these efforts, success in device-level linking remains limited.

Contrastive learning has emerged as a promising technique for device linking. It enables models to learn discriminative feature representations by training on both similar and dissimilar image pairs. This approach is particularly effective in distinguishing between images captured by different cameras and those taken by the same device.

Reference [53] first discussed the use of a Siamese network to extract a camera model fingerprint, known as Noiseprint. As shown in Figure 3b, the Siamese network comprises two identical CNNs with a shared architecture and weights. It is trained using pairs of image patches from the same camera (label +1) or different cameras (label −1). For positive examples, weights are updated to reduce the distance between the outputs, while for negative examples, weights are updated to increase the distance.

In the same year, another Siamese network model was proposed with end-to-end training in [54]. This model, known as the forensic similarity network, comprises a CNN-based feature extractor and a three-layer similarity network. The feature extractor aims to extract forensic traces from two images, while the similarity network produces a score indicating if the forensic traces are consistent.

Later, a multi-layer perceptron network was introduced for feature extraction. It also adopted Siamese architecture with contrastive loss and logistic prediction [55]. As reported in [53,54,55], these networks were effective for model-level camera linking but not for device-level linking. In 2025, a new approach was proposed [56] that also used a Siamese network to extract the noise residuals. However, this method further refined the noise residuals using contextual information aggregation operations. These operations were designed to suppress scene content interference and enhance the extraction of device-level forensic features, rather than model-level features. For the first time, device-level linking between two images was successfully demonstrated under a deep learning framework.

**Table 4:** Chronological overview of deep learning techniques for device linking.

Year	Techniques	References
2019	Contrastive learning	Siamese network [53]
	Contrastive learning	Forensic similarity [54]
2020	Contrastive learning	Multi-layer with Siamese [55]
2022	Few-shot learning	Global fuzzification with information diffusion [50]
	Few-shot learning	Ensemble strategy [51]
2023	Few-shot learning	Coordinate pseudo-label selection [52]
2025	Contrastive learning	SiamNet with contextual information aggregation [56]

## 5. Performance Comparison and Benchmarking

### 5.1. Dataset

To evaluate the effectiveness of source camera identification and device linking methods, it is essential to benchmark them across diverse datasets and scenarios. In forensic applications, datasets should reflect real-world conditions, including variations in camera models and devices, image content, and post-processing effects. Table 5 provides an overview of datasets commonly used in image forensics research. These datasets are broadly categorized based on the nature of the images they contain: images captured under default settings, compressed images, and manipulated images. Note that all these datasets primarily consist of RGB images in JPEG format, as it is the most common format used by digital cameras and mobile devices.

Among them, the Dresden image database is one of the most widely used datasets for both source camera identification and device linking [57]. It consists of 16,960 full-size photographs captured by seventy-four devices across twenty-five camera models from fourteen different camera brands. This dataset serves as a standard benchmark for most algorithms in this domain. The VISION dataset was introduced in 2017 [63]. It includes data that has undergone compression to simulate real-world sharing scenarios. The dataset contains 34,427 images and 1914 videos from thirty-five devices across eleven camera brands. It includes both original and social media compressed versions (from platforms like Facebook, YouTube, and WhatsApp). Flickr is a popular image-sharing platform; some researchers have collected images from it for testing [65,66]. Between 2018 and 2023, several datasets were introduced for source camera identification [58,59,60,61,62,64]. However, most existing methods have yet to be evaluated using these datasets.

Other datasets, such as CASIA and DF2023, focus on image manipulation (e.g., copy-move, splicing, enhancement) [67,68,69]. While valuable for evaluating forgery detection algorithms, they are not suitable for source camera identification due to missing camera information. An exception is the seam carving manipulation datasets [26,70], which retain crucial source camera information and can be used to assess the robustness of identification methods under content-aware image alterations.

DL-based methods adopt a classification setting, where the outputs represent scores indicating the likelihood that a test photo belongs to each camera class. Standard evaluation metrics for performance assessment include true-positive rate, false-positive rate, accuracy, precision, and F1 score.

### 5.2. Performance of Deep Learning Methods in Source Camera Identification

Table 6 summarizes the performance of deep learning-based methods for source camera identification at both the model and device levels. The Dresden [57] and VISION datasets [63] are among the most widely used benchmark datasets in the literature for evaluating source camera identification methods. We can also visualize the performance in accuracy over the years for different experimental setups.

**Figure 4:**
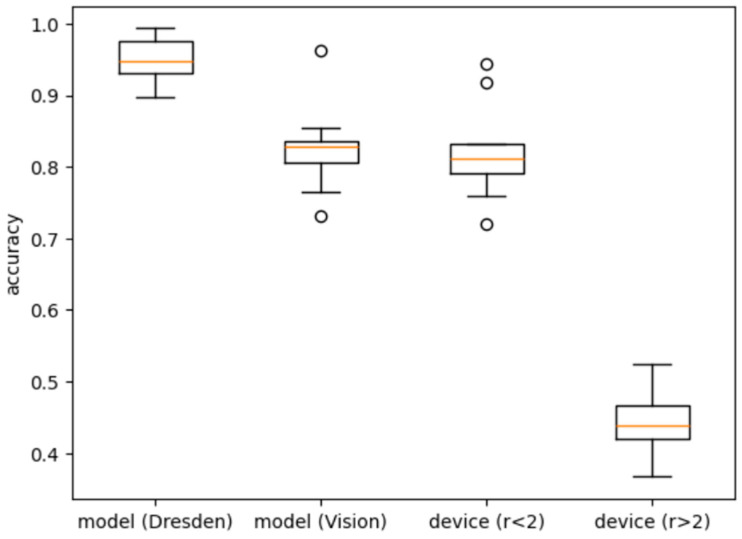
Comparison of the model-level and device-level accuracies in deep learning-based methods for source camera identification, where r denotes the average number of devices per model. The orange color lines represent the median.

By examining Table 6a, it is evident that all methods demonstrate strong performance in model-level camera identification, with accuracies ranging from 0.73 to 1.00, and an average of 0.92. As illustrated in the boxplot in Figure 4, model-level identification on the Dresden dataset surpasses that on the Vision dataset. This can be attributed to the more complex and diverse scenes present in the Vision dataset, which pose greater challenges for classification.

However, the transition to device-level identification results in a notable drop in performance, as evident in both Table 6b and Figure 4. Generally, as the average number of devices per camera model increases, identification accuracy tends to decline. When the average number of devices per model is below 2, the average accuracy remains around 0.82. In contrast, when this number exceeds 2, the average accuracy drops to 0.44, underscoring the difficulty of distinguishing individual camera devices. This limitation stands in comparison to traditional PRNU-based methods, which consistently achieve over 0.9 in device-level identification (as shown in Table 1).

To enhance device-level performance, a separate network for extracting noise residues from each camera was considered in [71]. Although this approach improves device-level accuracy, it is time-consuming and impractical given the large number of camera devices in real-world applications. In fact, PRNU-based methods are computationally lightweight, relying on correlation measures and filtering. DL-based methods generally require substantial resources for training (e.g., GPUs and large datasets). Despite these higher computational costs, they still fail to outperform PRNU at device-level identification. This raises questions about the cost-benefit trade-off for practical deployment.

### 5.3. Performance of Deep Learning Methods in Device Linking

This section evaluates the performance of deep learning methods for device linking tasks, focusing on two main strategies: one-shot learning and contrastive learning. Table 7 summarizes the results at both the model-level and device-level linking. Generally, one-shot learning strategies show limited performance. At the model-level, accuracy is around 0.55, while device-level accuracy drops further to 0.38, indicating challenges in reliably linking images from the same device.

Contrastive learning methods have shown more promise in device linking tasks. At the model level, the average accuracy is 0.69, which is lower than the model-level source camera identification performance shown in Table 7b and Figure 4. However, for device-level performance, the accuracy is around 0.65, which is comparable to the device-level source camera identification performance as in Table 7b and Figure 4.

Among the reviewed methods, the approach in [56] stands out for its device-level performance. Evaluated on an imbalanced dataset of 30 devices from 9 camera models, one should consider the F1 score as well. Its F1 score is 0.82, outperforming earlier methods. For instance, the U-Net-based approach [39] achieved an F1 score of 0.22, which is comparable to the performance of PRNU-based methods, as shown in Table 2. The forensic similarity network [54] had an F1 score of 0.12. These results demonstrate that device-level linking between two images is possible under a contrastive learning framework.

**Figure 5:**
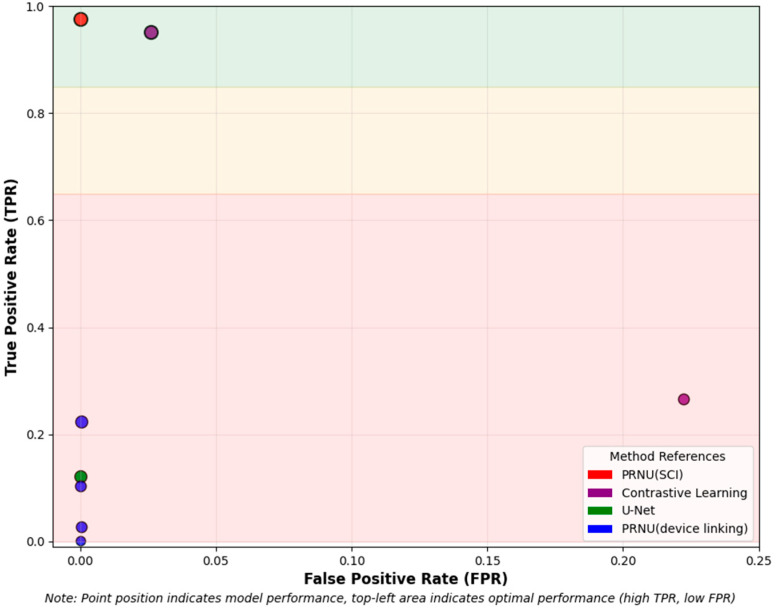
A plot of true-positive rate against false-positive rate for PRNU-based device linking methods [10,13,14,15] (blue), U-Net [39] (green), and contrastive learning methods [54,56] (purple). The red color represents the performance of the PRNU-based method [21] in a source camera identification setting where enough images are used to construct the camera PRNU.

We also consider metrics such as true-positive rate and false-positive rate, in addition to the F1 score. Figure 5 shows a plot of true-positive rates against false-positive rates. The blue markers represent PRNU-based methods for device linking, while the green markers correspond to the U-Net approach [39]. Both exhibit low true-positive rates, despite achieving excellent false-positive rates. This indicates that while they rarely link images from different cameras, they fail to link images from the same device correctly. The purple markers denote contrastive learning-based methods. Among these, the method in [54] achieves a higher true-positive rate, but at the same time, a high false-positive rate. In contrast, the method in [56] demonstrates an excellent true-positive rate, though its false-positive rate remains relatively high, which may limit its forensic applicability. The red markers illustrate the performance of PRNU-based methods for source camera identification, where enough images are available to construct a reliable camera fingerprint. Although the method in [56] has only two images for comparison and lacks a reliable camera fingerprint, it achieves a comparable true-positive rate to the traditional PRNU-based approach. However, the high false-positive rate highlights a critical challenge; further research is needed to reduce the false positives and enhance their reliability in practical forensic scenarios.

## 6. Discussions and Challenges

### 6.1. Device-Level Performance

Despite significant progress in DL-based methods for source camera identification and device linking, their performance at the device level remains far below that of PRNU-based methods. PRNU methods consistently achieve high accuracy and low false-positive rates, making them legally accepted benchmarks. In contrast, DL-based methods struggle to extract device-specific artefacts. This gap highlights the need for additional research to achieve device-level performance comparable to that of PRNU-based methods.

Further work can focus on developing strategies that capture device-specific forensic traces, such as using attention mechanisms [72] to direct the network’s focus toward camera-specific features while suppressing scene content. A contrastive learning framework [73] can be utilized to integrate information across various domains and at multiple scales, thereby enhancing device feature learning and characterization.

### 6.2. Cross-Dataset Validation

Forensic applications involve factors such as social media compression, varying image resolutions, and cameras not presented in the training data. While current DL-based methods perform well on benchmark datasets like Dresden or VISION, their accuracy on images from cameras outside the training set remains uncertain. These benchmarks also do not fully capture real-world diversity, leading to weak generalization to unseen cameras and datasets. To address this limitation, cross-dataset validation is essential. This process involves evaluating models on datasets that differ from the training set to assess robustness and prevent overfitting to specific benchmarks [74]. Such validation ensures that algorithms can handle forensic scenarios involving unknown devices and diverse imaging conditions.

### 6.3. Computational Costs

Given the large number of camera devices, computational efficiency is critical for practical deployment. Most existing studies emphasize classification performance and do not report efficiency metrics. Deep learning-based models typically demand substantial resources for training, including high-performance GPUs and large datasets, making them more resource-intensive than PRNU-based methods. Future studies could consider developing lightweight architectures, such as teacher–student frameworks [75], to reduce computational requirements and make DL-based solutions more feasible for forensic applications.

### 6.4. Modern Imaging Pipeline

Nowadays, enhancements to image quality can occur during the image capture process. Modern cameras are equipped with automatic settings, such as scene recognition, object detection, and face identification, to optimize photographs in real-time [76,77,78]. Additionally, popular mobile applications offer beautification options, including skin smoothing and blemish removal, during the photo capture process. However, existing datasets rarely account for these enhancements, making this an underexplored area of research. As noted in [7], the reliability of PRNU as the gold standard in modern imaging pipelines has been questioned due to such processing. In this part, we investigate whether these built-in enhancements would influence the performance of DL-based source camera identification or device linking, as observed with PRNU-based methods.

**Figure 6:**
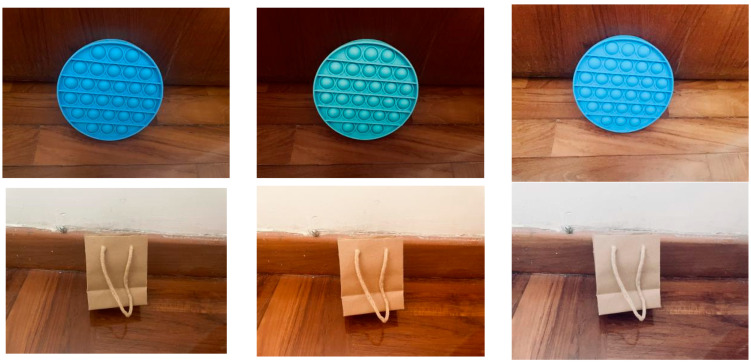
Example images considered in the robustness test. The first column displays the original images captured using the default camera settings. The second and third columns show images in scenario 1 (S1) and scenario 2 (S2), respectively.

**Figure 7:**
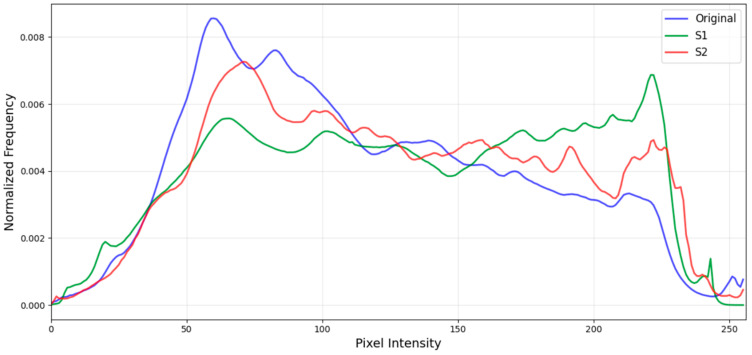
The average intensity histogram of the original images captured using the default camera settings: images in scenario 1 (S1) and scenario 2 (S2).

Two kinds of common photography scenarios are considered. They are camera-built-in image enhancement (S1) and AI enhancement via apps (S2). The former involves applying software-built-in processing through various kinds of settings to enhance the quality of photographs, while the latter employs specific apps that offer beautification features. Figure 6 shows some examples. In the first scenario (S1), the camera is set to enhance its vividness. In the second scenario (S2), an app named “Meitu” is used for AI-based enhancements, including “whitening”, “face whitening”, and “face skimming”. The average intensity histograms are shown in Figure 7. Overall, the histograms exhibit a similar trend; however, S1 and S2 display higher peaks at intensity levels around 220, likely due to whitening processes and the enhancement of vividness. The mean intensities for S1 and S2 are 132 and 127, respectively, which are higher than the average intensity of 115 observed in images captured at default settings.

Three methods were examined to evaluate their performance on these two scenarios: the PRNU-based method (Section 2), the deep learning method for source camera identification (Section 3), and the deep learning method for device linking (Section 4).

**Figure 8:**
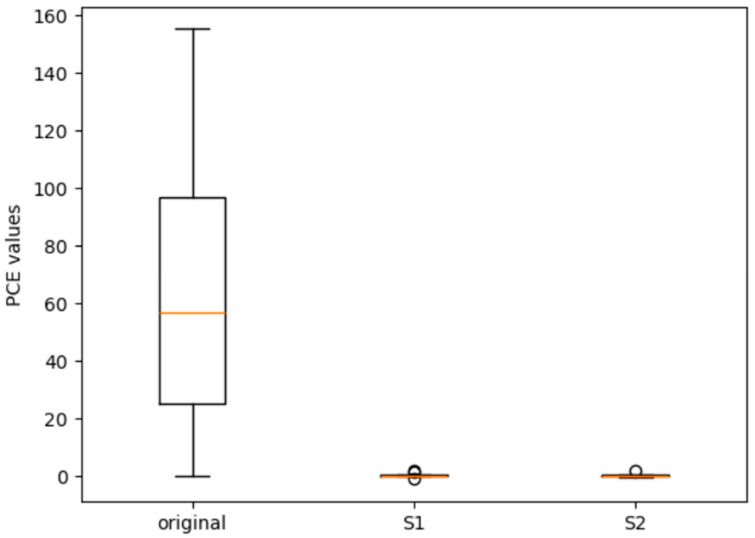
Comparison of the *PCE* values of the PRNU-based methods for original images: enhanced images in scenario 1 (S1) and scenario 2 (S2). The orangle line represents the median.

For the PRNU-based algorithm, the filter used is the wavelet-based denoising filter, and the top left corner was chosen for source identification, following common practice to minimize textured regions [2]. Figure 8 shows the box plot for *PCE* values of the PRNU-based methods in the two scenarios. A large *PCE* value suggests that the images are highly likely to have been taken from the specific camera device. Using the default setting, the average *PCE* value is 63.37. Out of 15 images, 13 have *PCE* values greater than 20. However, after using software-built-in processing or AI enhancement, the average *PCE* values for both scenarios drop significantly. They decrease to 0.15 and 0.27 for the two scenarios, respectively. The highest *PCE* value is 2.06, far below the normal threshold value for source camera identification and device linking applications. This finding aligns with the descriptions in [7], indicating that PRNU-based methods struggle to identify camera devices, even with the minor enhancements evaluated in the two scenarios.

After confirming the effect on PRNU methods, we investigated the impact of the deep learning technique on source camera identification. Specifically, a CNN method with good model-level performance was examined [34]. In the original case without any enhancement, 14 out of 15 images were successfully identified. In scenarios S1 and S2, 13 out of 15 images were accurately identified. Compared to PRNU-based methods, the CNN method demonstrates greater robustness. This resilience can be attributed to the CNN method, which primarily extracts model-based artefacts, which are less susceptible to alterations compared to device-based artefacts.

**Figure 9:**
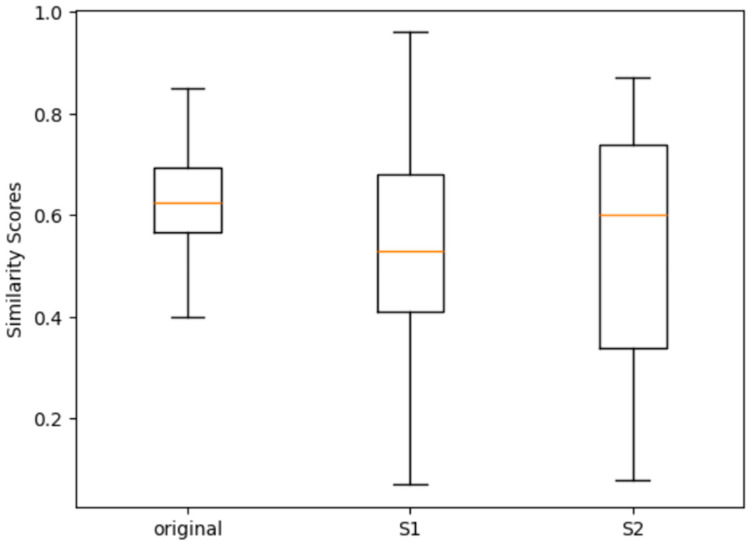
Comparison of the similarity scores of the Siamese network method for original images: enhanced images in scenario 1 (S1) and scenario 2 (S2). The orange line represents the median.

We then consider the effect on device linking applications. The SiamNet was examined due to its good device-level performance [56]. Figure 9 shows the similarity scores for the original case and the two scenarios. Using the default setting without any software-built-in-processing, the average similarity score is 0.627. With minor enhancement, the average similarity scores for the two scenarios are 0.524 and 0.536, respectively. With a threshold of 0.5, the average accuracy is 88.9% in the original case. However, the accuracy decreased to 60.5% and 58.0% for the two scenarios, indicating significant performance drops in both cases.

In summary, our findings indicate that, like PRNU-based methods [7], DL-based methods are also affected by the built-in processing features embedded in cameras. This underscores the need for updated datasets that reflect current imaging trends, including camera-integrated processing and AI-based enhancement, to strengthen the robustness of forensic methods against challenges posed by evolving practical photography practices.

## 7. Conclusions

Source camera identification and device linking are critical tasks in digital forensics, security, and online authentication. Over the years, a wide range of deep learning architectures have been explored to address these challenges. From early convolutional neural networks to advanced encoder–decoder structures, residual networks, dual-branch models, and contrastive learning frameworks, researchers have developed numerous sophisticated methods to extract forensic features from images. These deep learning approaches have shown strong performance in source camera identification, particularly at the model level. However, achieving reliable device-level identification remains a significant challenge due to the subtle nature of device-specific artefacts. Recent contrastive learning methods have demonstrated promising results in device linking, achieving higher F1 scores than earlier DL-based methods; however, they still suffer from higher false-positive rates compared to PRNU-based methods.

Recently, PRNU-based methods have been found to have limitations when confronted with modern image capture practices, such as built-in camera enhancements and AI-based image processing. Our findings reveal that DL-based methods also suffer from this issue. This underscores the need for continued innovation and updated datasets that reflect real-world conditions to improve the resilience of forensic techniques.

## Figures and Tables

**Table 1:** Performance of the PRNU-based source device identification methods for 19 camera devices in the Dresden dataset.

References	Accuracy	True-Positive Rate at a False-Positive Rate of 10^−3^
2009/2013, [2,13]	0.9032	0.7768
2009, [15]	0.9116	0.7674
2012, [16]	0.9000	0.7672
2017, [17]	0.9263	0.8011

**Table 2:** Performance of the sensor noise-based methods in the device linking setting for 30 camera devices in the Dresden dataset.

References	True Positive Rate	False Positive Rate	Accuracy	F1 Score
2009, [15]	0.0013	0.0002	0.9354	0
2013, [13]	0.1040	0.0002	0.9420	0.19
2019, [14]	0.2240	0.0004	0.9496	0.36
2021, [10]	0.0267	0.0004	0.9369	0.05

**Table 5:** Datasets for image forensics applications.

Nature of Images	References	Dataset Name	Number of Images	Number of Camera Devices	Number of Camera Models
Images acquired under default settings	[57]	Dresden	16,960	74	25
	[58]	MICHE-I	3700	3	3
	[59]	UNIFI	5415	23	21
	[60]	IMAGINE	2816	67	55
	[61]	SOCRatES	9700	103	65
	[62]	Daxing	43,400	90	22
Compressed images at different qualities	[63]	VISION	34,427	35	29
	[64]	Forchheim	23,000	27	25
	[65]	---	13,210	400	---
	[66]	---	32,445	486	---
Forged images (copy move, splicing, enhancement)	[67]	CASIA	5123	No camera information	
Forged images (copy move, splicing, removal, enhancement)	[68]	IMD2020	2010	No camera information	
	[69]	DF2023	1 million	No camera information	
Forged images (seam carving)	[26]	---	2750	11	10
	[70]	---	1560	13	12

**Table 6:** (**a**) The model-level and (**b**) device-level performance of deep learning-based methods for source camera identification.

**(a)**		
Number of Camera Models (Dataset)	References	Accuracy
25 models (Forchheim) (selecting 50 patches)	2025, [49]	0.9673 (F1: 0.9604)
	2024, [47]	0.9451 (F1: 0.9312)
	2024, [45]	0.9413 (F1: 0.9266)
	2021, [43]	0.9387 (F1:0.9035)
	2021, [34]	0.9497 (F1: 0.9475)
	2021, [42]	0.9105 (F1: 0.9139)
	2017, [31]	0.8717 (F1: 0.8667)
	2017, [32]	0.8833 (F1: 0.8924)
25 models (Forchheim) (selecting 256 image patches)	2024, [47]	1.000
	2021, [34]	0.9987
	2021, [42]	0.9948
	2021, [43]	0.9961
	2017, [3]	0.9361
23 models (Dresden)	2024, [46]	0.9933
	2021, [42]	0.9862
	2021, [43]	0.9851
	2017, [31]	0.9806
	2017, [36]	0.9735
18 models (Dresden)	2025, [49]	0.957 (F1: 0.951)
	2025, [48]	0.950 (F1: 0.945)
	2024, [45]	0.938 (F1: 0.931)
	2024, [47]	0.944 (F1: 0.939)
	2022, [44]	0.931 (F1: 0.932)
	2021, [34]	0.942 (F1: 0.931)
	2021, [42]	0.912 (F1: 0.904)
	2021, [43]	0.932 (F1: 0.932)
	2019, [53]	0.913 (F1: 0.916)
	2017, [32]	0.898 (F1: 0.891)
13 models (Dresden)	2023, [40]	0.9760 (F1: 0.9759)
	2019, [71]	0.9756 (F1: 0.9760)
	2017, [3]	0.9034 (F1: 0.9050)
4 models (Dresden)	2024, [38]	0.9570
29 models (Vision)	2025, [49]	0.891 (F1: 0.920)
	2025, [48]	0.855 (F1: 0.893)
	2024, [45]	0.831 (F1: 0.876)
	2024, [47]	0.837 (F1: 0.878)
	2022, [44]	0.823 (F1: 0.862)
	2021, [34]	0.829 (F1: 0.867)
	2021, [42]	0.765 (F1: 0.792)
	2021, [43]	0.826 (F1: 0.872)
	2019, [53]	0.801 (F1: 0.813)
	2017, [32]	0.732 (F1: 0.794)
4 models (Vision)	2024, [38]	0.9629
15 models (from [41])	2024, [41]	0.9787
	2021, [43]	0.9234
	2019, [37]	0.9509
	2017, [36]	0.9356
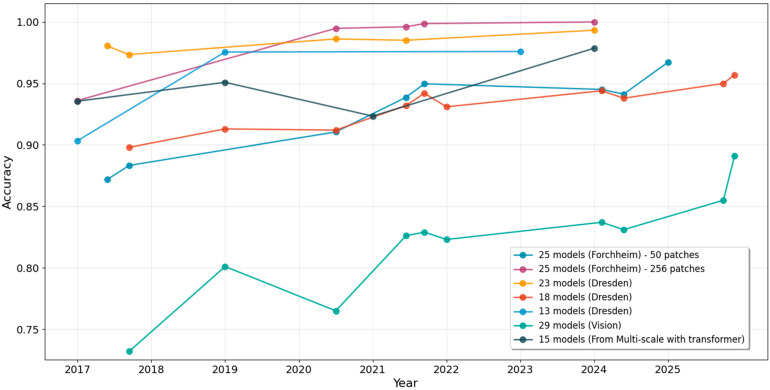		
**(b)**		
Number of Camera Devices (Dataset)	References	Accuracy
74 devices (Dresden) Average no of devices per model = 2.96	2025, [48]	0.492 (F1: 0.486)
	2024, [45]	0.414 (F1: 0.416)
	2024, [47]	0.475 (F1: 0.471)
	2022, [44]	0.439 (F1: 0.448)
	2021, [34]	0.446 (F1: 0.467)
	2021, [42]	0.393 (F1: 0.393)
	2021, [43]	0.428 (F1: 0.446)
	2019, [37]	0.5240
	2019, [53]	0.427 (F1: 0.444)
	2017, [31]	0.4581
	2017, [32]	0.367 (F1: 0.392)
35 devices (vision) Average no of devices per model = 1.21	2025, [35]	0.943
	2024, [45]	0.813
	2022, [39]	0.811
	2022, [44]	0.832
	2021, [42]	0.721
	2021, [43]	0.765
	2019, [71]	0.831
	2017, [32]	0.830
13 devices (from [41]) Average no of devices per model = 1.625	2024, [41]	0.9185
	2021, [43]	0.7996
	2019, [36]	0.8023
	2017, [37]	0.7585
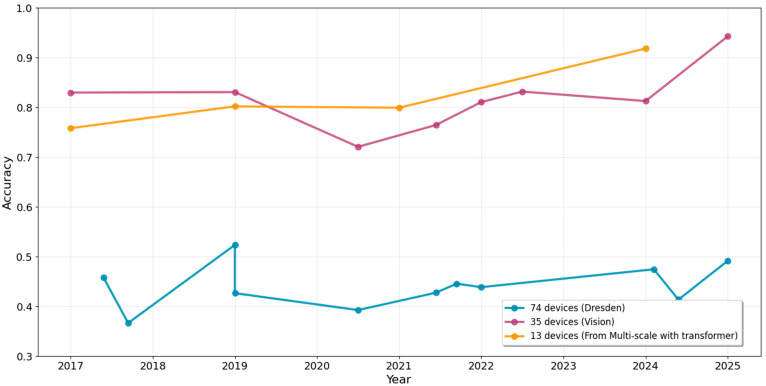		

**Table 7:** The performance of (**a**) one-shot learning and (**b**) contrastive learning for device linking [52].

**(a)**			
**Linking**	**Number of Cameras (Dataset)**	**Accuracy**	
Model-level	14 models (Dresden)	0.5660	
	11 models (Vision)	0.5303	
Device-level	27 devices (Dresden)	Around 0.4	
	35 devices (vision)	around 0.35	
**(b)**			
**Linking**	**Number of Cameras (Dataset)**	**References**	**Accuracy**
Model-level	5 models (Dresden)	2020, [55]	0.7840
		2017, [3]	0.6930
		2016, [30]	0.6530
	5 models (Vision)	2020, [55]	0.7450
		2017, [3]	0.6250
		2016, [30]	0.6380
Device-level	30 devices (Dresden) Average number of devices per model = 3.33	2025, [56]	0.9520 (F1 = 0.82)
		2022, [39]	0.9431 (F1 = 0.22)
		2019, [54]	0.7448 (F1 = 0.12)
	5 devices (Dresden) from one camera model	2020, [55]	0.6530
		2017, [3]	0.6863
		2016, [30]	0.6397
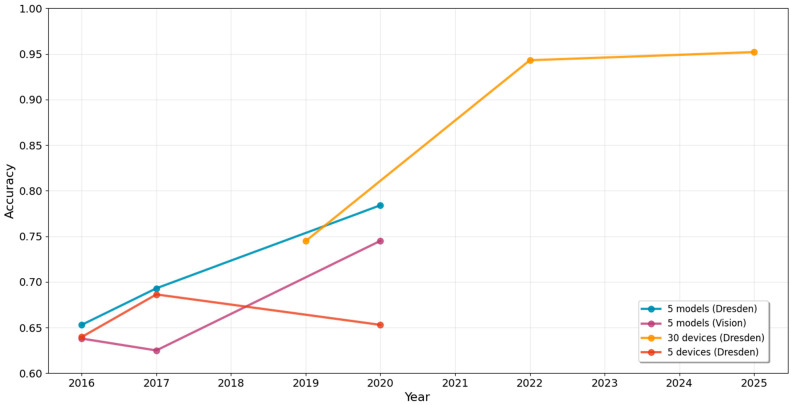			

## Data Availability

No new data were created or analyzed in this study. Data sharing is not applicable to this article.

## Abbreviations

The following abbreviations are used in this manuscript:

CNN	Convolutional neural networks
DL	Deep learning
*PCE*	Peak correlation energy
PRNU	Photo-response non-uniformity
SVM	Support vector machine
